# Supplementary material for: Are carbon emissions trading and green financial instruments synergistic? -Comprehensive quantitative research based on content analysis
Source: PLoS One. 2024 Mar 7;19(3):e0298601. doi: 10.1371/journal.pone.0298601 (PMC10919601; doi:10.1371/journal.pone.0298601)
Supplement: S1 Data — (ZIP) [file pone.0298601.s001.zip › Data/地级市Z_F工作报告.zip!/╣π╢1⁄2/╔ε█┌╩╨/└┤╫╘╣1⁄2╓┌║┼í╛┬φ┐╦╩2╛▌═°í┐_2019.docx]

各位代表：

　　现在，我代表深圳市人民政府向大会作政府工作报告，请予审议，并请各位政协委员和其他列席人员提出意见。

　　一、2018年工作回顾

　　过去一年是改革开放40周年，是深圳改革发展史上具有重要意义的一年。习近平总书记亲临广东、深圳视察，并专门对深圳工作作出重要批示指示，充分肯定深圳各项事业发展取得显著成绩，要求深圳朝着建设中国特色社会主义先行示范区的方向前行，努力创建社会主义现代化强国的城市范例，在新时代走在前列、新征程勇当尖兵。习近平总书记的一系列重要批示指示，赋予了深圳新时代发展的新定位、新使命，极大地鼓舞了全市人民，增添了我们改革开放再出发的强劲动力。

　　一年来，我们把学习贯彻习近平新时代中国特色社会主义思想和党的十九大精神作为头等大事和首要政治任务，全面落实习近平总书记对广东重要讲话和对深圳重要批示指示精神，树牢“四个意识”，坚定“四个自信”，坚决做到“两个维护”，落实党中央国务院、省委省政府和市委的部署，坚持稳中求进工作总基调，践行高质量发展要求，全面推动改革开放创新，深入开展“城市质量提升年”工作，较好地完成了市六届人大六次会议确定的目标任务。

　　——经济运行稳中有进、质量更好。初步核算，2018年本市生产总值突破2.4万亿元、同比增长7.6%，经济总量居亚洲城市前五；辖区公共财政收入9102.4亿元、增长5.5%；地方一般公共预算收入3538.4亿元、增长6.2%；居民人均可支配收入增长8.7%。经济发展有速度、有质量、有效益。

　　——以营商环境改革为重点的全面深化改革取得重大进展。在全国率先改革并实施多层次住房供应保障制度，营商环境改革、工程建设项目审批“深圳90”改革、智慧城市和数字政府建设等走在全国前列。前海蛇口自贸片区一批制度创新成果在全国复制推广。深圳成为全国营商环境最好的城市之一。

　　——高新技术产业发展成为全国的一面旗帜。全社会研发投入占GDP比重、PCT国际专利申请量全国领先，国家级高新技术企业数量居全国第二，数字经济发展走在全国前列。光明科学城、鹏城实验室、深圳湾实验室等重大创新平台启动建设。深圳获批国家可持续发展议程创新示范区，成为中国最具创新力的城市，在全球创新体系中的地位不断提升。

　　——高水平推进枢纽型城市建设取得新突破。粤港澳大湾区建设提速，广深港高铁开通运营，轨道交通建设加快，机场、港口、高铁枢纽功能进一步强化，国际航线大幅增加，国际航空旅客增幅全国第一。重点片区规划全面上水平。深圳跻身世界一线城市行列，入选全球十大最佳旅行目的地。

　　——天更蓝、地更绿、水更清、城市更加和美宜居。城市重要节点、主干道、城中村等环境品质显著改善。90%的黑臭水体得到治理、基本实现不黑不臭。PM2.5平均浓度降至26微克/立方米，空气优良率创近年来新高，空气质量在全国169个重点城市中排名第六。深圳蓝、深圳绿、辉煌新时代灯光夜景成为城市的亮丽名片。深圳以更加优美的城市面貌庆祝改革开放40周年。

　　——以人民为中心的发展让市民更有获得感、幸福感、安全感。多年未解决的中小学校内午餐午休问题得到较好解决，一批底子差的幼儿园办学条件明显改善。基础教育学位大幅增加，高考成绩再创新高。一批高水平大学、高水平医院落地，新十大文化设施、重大民政设施等规划建设加快启动。九大类40项民生实事基本按计划完成，最低工资标准、最低生活保障标准逐步提高。成功抗击深圳35年以来最强台风“山竹”，治安警情大幅下降，社会建设和社会治理现代化水平上了新台阶。深圳成为全国最安全稳定的城市之一。

　　一年来，我们主要做了以下工作：

　　（一）推动实体经济稳健发展。我们积极应对复杂多变的外部环境，坚持以供给侧结构性改革为主线，及时推出“一揽子”惠企政策措施，保持了实体经济发展的良好势头。一是精准施策支持企业发展。推出促进民营经济发展“四个千亿”措施，全年为企业减负超过1400亿元，民营经济增加值增长9.6%。率先出台化解上市公司流动性风险的政策措施，在全国设立首个150亿元的政策性纾困资金。着力破解中小微企业融资难问题，组建30亿元的政策性融资担保基金，设立50亿元的银行贷款风险补偿资金池，全年小微企业贷款余额增长16%。降低实体经济用电成本，在落实国家统一降电价基础上，对先进制造业和工商业用电成本分别再降20%和10%，企业对惠企政策有更多实实在在的获得感。二是着力提升先进制造业竞争力。工业投资增长8%以上，工业技改投资增长超过20%。华星光电T6项目投产、T7项目开工，柔宇柔性显示、开沃新能源汽车等重大项目建成，安谋中国、中国移动智能硬件创新中心等一批高端项目落地，新增石墨烯等省级制造业创新中心4家。全年规模以上工业增加值增长9.5%，先进制造业增加值增长12%，二产占GDP比重保持在40%以上。以高端制造业为核心的实体经济发展根基更加稳固，经济结构持续优化。三是全力拓展产业发展空间。出台工业区块线管理办法，划定270平方公里工业用地红线。深入实施拓空间十大行动，完成旧工业区综合整治140万平方米，城市更新供应用地2.57平方公里，整备土地13平方公里，其中较大连片土地5.4平方公里。全面开展查违三年攻坚行动，出台产业类和公共配套类历史违建处理办法，拆除消化违法建设超过2100万平方米。节约集约用地、盘活土地存量又有新业绩。四是促进投资消费出口均衡发展。采取一系列措施扩投资、促消费、稳外贸。全年固定资产投资6191亿元、增长20.6%；社会消费品零售总额6169亿元、增长7.6%；进出口总额3万亿元、增长7%，跨境电商、平行汽车进口等外贸新业态快速增长。

　　（二）推动重点领域改革再突破。充分发挥先行先试作用，着力抓好115项国家级改革试点，努力向改革要活力。一是率先深化营商环境改革。出台营商环境改革20条政策措施，企业开办时间从9天缩短至4天，出口通关、外商登记备案时间分别压缩69%、60%，企业出口退税时间压缩20%，12项不动产登记业务实现一小时办结，政府投资项目从立项到施工许可审批由原来的267个工作日减少到90个工作日以内，社会投资项目控制在33个工作日以内。营商环境的优化，进一步增强了创新创业活力，全年新设立企业29万户、总量增长11.6%。新增上市公司16家，新引进持牌金融机构26家。投资发展、创新创业氛围日益浓厚。二是率先推进住房制度改革。出台全国首个关于加快建立多主体供给多渠道保障租购并举的住房供应与保障体系的意见，构建商品房占40%、政策性支持住房占40%、公租房占20%的供应结构，着力解决住有所居的问题，破解高房价对实体经济和人才的挤压影响。全年建设筹集人才住房和保障性住房8.9万套、供应4.6万套，开工老旧小区改造7890套。稳房价、稳租金、稳预期取得实质进展。三是持续深化科技管理体制改革。在全国率先实施重大科技项目评审专家“主审制”，出台科研项目经费管理改革20条，赋予高校、科研院所更大的科技经费管理自主权。改革科研资金、战略性新兴产业资金等使用管理办法，形成事前竞争立项、事后奖励等多元化支持方式，规范科技经费和产业资金使用、堵塞腐败漏洞有新举措。四是全面推进国企、医疗等领域改革。获批开展全国区域性国资国企综合改革试验，实施国企混改三年行动。罗湖医改、药品集团采购改革经验在全国推广。生态文明体制改革扎实推进，罗湖、坪山和大鹏获评国家生态文明建设示范区。文艺院团改革加快，成立深圳歌剧舞剧院和交响乐发展基金会。财政预算管理、税收征管体制、土地管理、社会组织管理等领域改革取得积极进展。国务院批复设立光明行政区，北部中心建设进入新阶段。

　　（三）全力落实粤港澳大湾区发展战略。制定贯彻落实粤港澳大湾区发展规划纲要的实施意见和三年行动方案，着力深化深港澳合作，支持港澳融入国家发展大局，推动形成全面开放新格局。一是前海发展生机勃勃。完成前海城市新中心规划优化，实施城市新中心建设三年行动计划，突出产城融合、减量提质、山海统筹、宜居优先，推进功能与环境“双提升”工程，开工妈湾跨海通道等重大项目。开展深港合作专项行动，港企总数突破1万家、对前海税收贡献近1/4，港交所前海联合交易中心开业，新华养老保险等一批总部项目落户，深港基金小镇投入使用，前海青年梦工场新引进港澳青年创业团队31个。出台加快前海开发开放高质量发展的措施，11项制度创新成果在全国复制推广。前海开发开放成效得到习近平总书记充分肯定。二是深港澳合作更加紧密。深港澳常态化联络机制不断完善，港澳人士特别是青年在深圳工作生活更加便利。河套深港科技创新合作区规划建设加快，往返香港科学园直通巴士开通，61个合作项目落地。西九龙站口岸“一地两检”顺利实施，莲塘口岸联检楼基本完工。深港国际中心动工建设。深澳创意周等系列文化交流活动成功举办，深澳中医药创新研究院等项目稳步推进。深港澳深度合作迈上新台阶。三是开放型经济水平持续提升。新设外商投资企业增长近1.2倍，实际利用外资82亿美元、增长11%，对外直接投资增长118%。对“一带一路”沿线市场进出口额增长9.9%，建成中国巴新友谊学校，加快推进巴新拉姆二期水电等项目，深越海防合作区一期工程完工。新增米兰、迪拜2个友好交流城市，新增伦敦、巴黎等15个国际通航城市，国际航空旅客增长34%，国际邮轮旅客增长93%。四是深汕特别合作区等建设进入新阶段。合作区正式揭牌，总体规划和城市设计基本完成，新开工重大产业项目10个，小漠国际物流港等项目加快推进。深莞惠“3＋2”经济圈合作不断深化，深圳与哈尔滨33个对口合作项目加快实施。

　　（四）着力壮大创新发展新动能。坚持抓创新就是抓发展、谋创新就是谋未来，积极构建“基础研究＋技术攻关＋成果产业化＋科技金融”的全过程创新生态链。一是做强做大新经济。出台加快高新技术产业高质量发展决定，制定加快发展战略性新兴产业实施方案，设立全国首个50亿元天使投资引导基金，全社会研发投入超过1000亿元，国家级高新技术企业预计新增3000家以上、总量超过1.4万家，战略性新兴产业增加值增长9.1%。深圳获批建设海洋经济发展示范区。二是加快提升自主创新能力。制定加强基础研究的实施办法，开展芯片、医疗器械等10项关键零部件重点技术攻关。开工合成生物研究、脑解析与脑模拟等重大科技基础设施，启动建设肿瘤化学基因组学国家重点实验室，新组建第三代半导体研究院等新型基础研究机构10家，新增各类创新载体189家。获科技进步一等奖等国家科技奖16项、中国专利金奖4项。专利授权量增长48.8%。科技进步对经济增长的贡献率进一步提升。三是持续优化创新环境。依法实施更严格的知识产权保护，中国（深圳）知识产权保护中心和南方运营中心正式挂牌。出台鹏城英才计划等政策，成立国家级人力资源服务产业园。全年新引进人才28.5万名、增长8.4%，新增全职院士12名、总量增长41%，新增高层次人才2678名、增长59%。

　　（五）集中力量打好三大攻坚战。采取超常规的举措、综合治理的办法，加快补齐高质量全面建成小康社会的短板。一是加强重点领域风险防控。持续开展互联网金融风险专项整治，建设灵鲲金融安全大数据监控平台，网贷平台等风险整治取得阶段性成效。继续落实房地产市场调控部署，适时补充出台调控措施，制定稳定住房租赁价格的意见，保持了房地产市场总体健康稳定发展。二是加大精准扶贫力度。全年实施对口帮扶6省34县项目225个，投入财政帮扶资金48亿元，惠及贫困人口近10万人。新疆喀什深圳产业园累计注册企业达522家，喀什大学新校区建成投入使用，深圳在全国对口援疆绩效考核中位居前列。建成西藏非物质文化遗产博物馆，开工建设察隅县人民医院、林芝华侨城国际汽车营地项目。深入推进广西百色、河池对口扶贫协作，深巴大健康合作特别试验区等一批项目按计划实施。对口帮扶河源、汕尾取得新进展，12个共建产业园区新引进亿元以上项目68个，深圳中学河源实验学校等项目启用。三是强力推进污染防治。中央环保督察“回头看”交办的526宗案件基本办结，完成了一级水源保护区1069栋违法建筑全部拆除任务。水污染治理力度空前，全面推行河长制、湖长制、湾长制。新建污水管网2855公里，基本补齐历史缺口。完成7146个小区雨污分流改造。新改扩建水质净化厂16座，新增污水处理能力66万吨/日。茅洲河污染指数下降47.3%，深圳河、观澜河、坪山河12月水质达到地表水Ⅴ类。国家海洋督察整改扎实推进，制定海洋环境保护规划，深圳湾综合污染指数下降32.5%。开展“利剑二号”环保执法行动，推行交叉执法，综合整治“散乱污危”企业9329家。实施“深圳蓝”可持续计划，落实工地扬尘治理7个100%等减排、控烟新举措，试行推广国VI标准车用燃油，淘汰老旧车约13万辆，新推广新能源汽车9万辆、新型智能全封闭式泥头车1500多辆，基本实现出租车纯电动化。新建充电桩2万个，增长68%。340座垃圾转运站完成升级改造，妈湾城市能源生态园建成调试，老虎坑三期、东部环保电厂项目按计划推进，预计今年6月投入使用。全市日产生活垃圾将基本实现焚烧处理。

　　（六）全面加强城市规划建设管理。瞄准建设全球一流城市，提高城市规划科学化、建设现代化、管理精细化和服务品质化水平。一是系统优化城市规划设计。高标准编制国土空间规划，完成新一轮对外交通规划布局和深圳港总体规划。出台加快建设全球海洋中心城市的决定和海岸带保护利用规划。编制重点片区城市设计工作方案和开发建设导则，制定打造城市建设精品若干措施，上线运行“多规合一”信息平台，推行重点区域总设计师负责制，开展城市设计国际咨询。深圳湾超级总部基地、香蜜湖新金融中心、海洋新城、光明科学城、大运新城、北站商务中心区等重点片区规划更具品位、更具特色。全市获国家优质工程奖和鲁班奖11项。二是加快完善现代化基础设施。成立基础设施投资基金，推行政府投资项目代建制，全年完成基础设施投资1419亿元、增长23.4%。机场卫星厅开工，赣深客专启动建设。轨道交通四期5条线路全面开工，在建里程达275公里。外环高速等加快推进，石岩外环路等8条道路建成通车。完成100个交通拥堵节点改造，打通红棉路、沙荷路等断头路20条，建成一批立体停车库，新建自行车道200公里。优化调整公交线路176条，公交分担率达60%，获评国家公交都市建设示范城市。三是持续提升城市环境品质。推进600个城中村综合治理，开展管道天然气入户百万工程。基本完成所有工地施工围挡改造提升。净化整治2730条道路城市家具，新建和改造提升1200多座公共厕所。新改扩建公园69个，建成花漾街区、街心花园184个，大沙河生态长廊等成为市民休闲的好去处。推进城市绿化净化美化亮化工程，世界著名花城、全国最干净城市建设取得新成效，荣获“国家森林城市”称号。全城展现出山清水秀、满眼绿色、花海缤纷、夜景璀璨、干净整洁、平安有序的新景象，城市美誉度显著提升。四是着力保障城市安全运行。推进城市公共安全风险排查治理，实施安全生产八大专项整治行动，重点整治道路交通、建筑施工、地下管线、电气火灾、电动单车违规行驶等突出安全隐患，交通事故起数下降13%、火灾起数下降26.7%，安全生产形势总体稳定。开展首次全市性防台风实战盲演和国防动员演练，城市应急管理水平和全社会防灾救灾能力得到强化提升。深入推进食品药品安全大排查、大整治，推进供深食品标准体系建设，实现食品安全监管“一街一车一室一中心一基地”全覆盖，全市没有发生重大食品药品安全事件。强力推进扫黑除恶专项斗争，打掉了一批涉黑团伙和“保护伞”。加强社会综合治理和治安巡逻防控，110接报刑事治安总警情下降23%，社会大局保持安全稳定。

　　（七）加快一流智慧城市和数字政府建设。制定新型智慧城市和数字政府建设总体方案，大力推广掌上政府、指尖服务、刷脸办事，努力让市民办事更方便、城市运行更高效。一是着力提高政府服务效率。上线“i深圳”统一政务服务APP，发布“不见面审批”、全城通办事项各300项，推出高校应届毕业生落户等45项“秒批”服务，养老金、工伤待遇实现“刷脸”领取，高龄老人津贴申请等高频事项实现“零跑动”。在市区两级政务办事大厅推出午间延时服务，新设置一批24小时智能自助办事终端，市民对政府便民服务的获得感明显提升。全面推广智能政务办公系统和视频会议，基本实现办公无纸化、移动化。清理行政职权事项164项，完成全市政务事项标准化梳理，行政审批时限大幅压缩，转变政府职能改革又有新成效。二是上线应用一批智慧民生服务。在全国首次成功实现5G网络传输4K超高清信号，初步建成政务信息资源共享平台和公共基础信息资源库。市属公立医院检查结果实现互认互通，居民电子健康卡率先启用，56项民生警务实现“零跑动”，公租房、安居型商品房水电气实现“一站式”办理，中小学入学网上一次申报全面推开。建立城市智能交通平台，实现公交地铁移动支付全覆盖。推出预约出行、停车指引等便民措施，智能城市交通服务又有新体验。三是加快推进智慧监管。推行“AI＋新警务”，率先对闯红灯等违法行为开展“刷脸”执法。推进大型客车、危险品运输车辆和泥头车等特种车辆智能监控全覆盖，动态监管疲劳驾驶、超速等违法行为，交通安全秩序进一步好转。全面高水平推动社区信息化平台建设，新安装城中村视频门禁系统3万余套，住宅小区和城中村管理服务智慧化取得新进展。推进智慧市场监管，实现企业信用和产品质量信息在线查询。食品药品溯源安全管理有了新手段。

　　（八）推动民生事业再上新水平。践行以人民为中心的发展思想，以更实举措补齐民生短板，让市民得到更多实惠。一是民生保障力度加大。全年九大类民生支出达到2772亿元，占一般公共预算支出的65%。完成民生微实事1.2万件。新增就业10.9万人，城镇登记失业率2.5%以内，最低工资标准提高到2200元/月，最低生活保障标准提高到1070元/月。二是教育事业发展提速。新增幼儿园学位2.3万个、公办中小学学位6.3万个，新增新型公办幼儿园150所、普惠性幼儿园116所，261所幼儿园完成托底改薄。75%的义务教育学校实现校内午餐午休。国家义务教育质量监测结果全省领先。深圳大学、南方科技大学等4所大学入选新一轮省高水平大学和学科建设计划，哈尔滨工业大学（深圳）单独招收本科生，中山大学深圳校区开工，深圳技术大学去筹设立，清华大学深圳国际研究生院获批，中科院深圳理工大学正式签约。深圳职业技术学院获国家级教学成果奖特等奖。三是医疗卫生事业加快发展。新增病床2555张，4家医院纳入省高水平医院建设计划，新增8家三甲和三级医院，市第三人民医院获批国家结核病临床医学研究中心，中山大学附属第七医院开业运营，新华医院、第二儿童医院开工，中国医科院阜外深圳医院落地。新引进高层次医学团队57个。新增社康机构41家，重点人群家庭医生签约率达到67%。开展母婴安全行动计划和健康儿童计划，免费实施适龄少儿水痘、流感疫苗接种和牙齿窝沟封闭等公共卫生服务。深圳健康养老学院成立，市养老护理院开业运营，新增养老床位1000张，“健康深圳”建设全面提速。四是文体事业繁荣发展。成功举办“大潮起珠江——广东改革开放40周年展览”、“追梦——改革开放再出发”晚会等重大庆祝活动，文博会、书博会、中国设计大展、深圳设计周暨环球设计大奖、“一带一路”国际音乐季、深圳读书月等活动擦亮城市文化品牌。当代艺术馆开馆，南山博物馆、龙岗三馆一城等建成使用。新建便民利民健身场所146个、社区体育公园12个。线上游客中心正式运营。深圳足球队晋级中超联赛，深圳运动员在第18届亚运会上取得4金5银3铜的优异成绩。获得WTA年终总决赛10年承办权，深圳国际马拉松、中国杯帆船赛等活动影响力不断扩大，城市文化软实力不断增强。

　　（九）进一步加强政府自身建设。坚决维护习近平总书记党中央的核心、全党的核心地位，坚决维护党中央权威和集中统一领导，扎实开展“大学习、深调研、真落实”工作，推动习近平新时代中国特色社会主义思想在深圳落地生根、结出丰硕成果。坚决执行市人大及其常委会决议决定，定期向市人大报告工作、向市政协通报情况，提请市人大审议法规草案17件，认真办理人大代表建议822件、政协提案536件，着力在提高办理质量、解决实际问题上下功夫，有力推动了垃圾分类、饮用水源保护等工作。落实法治中国示范城市建设实施纲要，制定政府规章13件，推行行政执法公示和全过程记录等制度，加强政务公开，深圳在100个大中城市法治政府评估中位列第一，预决算公开度排名全国第二。认真履行党风廉政建设主体责任，抓好中央巡视反馈意见整改落实，保持惩治腐败高压态势，严格落实中央八项规定及实施细则精神，进一步整治“四风”，全年压减“三公”经费14%。推动审计全覆盖向纵深发展，建立财政资金、国有资产交易、公共资源交易等信息化监管平台，廉政风险防范机制进一步健全。

　　此外，信访、统计、计生、民族、宗教、对台、侨务、民防、档案、史志、双拥等工作取得新进展。

　　各位代表，过去一年，我们面对复杂多变的外部环境，迎难而上、开拓进取，取得了来之不易的成绩。这是习近平新时代中国特色社会主义思想科学指导的结果，是以习近平同志为核心的党中央坚强领导的结果，是在省委省政府和市委的正确领导下，在市人大、市政协的大力支持和监督下，全市人民同舟共济、奋力拼搏的结果。在这里，我代表深圳市人民政府，向全市人民，向各位人大代表和政协委员，向各民主党派、各人民团体和各界人士，表示衷心的感谢！向中央和省各部门、中央和各省区市驻深单位，向驻深部队官兵，向长期以来关心和支持深圳发展的港澳同胞、台湾同胞、海外侨胞和国际友人，表示衷心的感谢！

　　在取得发展成绩的同时，我们也清醒地看到，深圳经济社会发展还面临很多问题和挑战，政府管理服务还没做得很好。特别是，经济稳增长压力仍然较大，国内外经济环境发生深刻变化，部分要素成本上涨较快，一些企业经营困难，产业整体竞争力还需提升，经济存在明显下行压力。自主创新能力有待增强，顶尖人才和高端研发团队缺乏，一些核心技术还没有做到真正自主可控，一些关键零部件和重大装备仍需依赖进口，基础研究水平还比较薄弱，源头创新能力还不足。城市治理还存在短板，城市建设管理标准化、精细化和智慧化程度不高，垃圾等固体废弃物处理问题依然突出，部分水体水质离地表水Ⅴ类还有差距，历史违法建设处置任务艰巨。个别市政工程存在过度改造、道路反复开挖、施工扰民等问题。生产安全、公共安全基础还不牢固。公共服务质量有待提高，优质教育、医疗等资源供给不足，高等教育总体水平还不高，高水平医院和高水平学科偏少，有的幼儿园办学水平偏低，一些社康中心条件比较薄弱，充电桩等便民设施建设滞后。政务服务的便利化、人性化程度不高，办事流程复杂、审批环节多、重复提交证明材料等问题还没有完全解决，有些办事指引不够清晰、咨询电话等待时间过长，少数窗口工作人员服务意识不强，市民办事体验还不够好。“放管服”改革还需深化，有的审批下放了、监管没跟上，有的不审批不监管、不审批不会管，基层执法中不同程度存在执法检查过多过频、标准不够明确、处罚“一刀切”等问题。政府信息的共享程度还不高，“信息孤岛”“数据烟囱”问题依然存在，数据归集使用标准不统一、质量不高、动态更新不及时，实现“让数据多跑路、群众少跑腿”还有很大空间。廉洁政府建设还需加强，部分政府采购、建设工程招投标存在围标串标、明招暗定等问题，有的国有资产存在“跑冒滴漏”，一些基层管理服务单位腐败问题不容忽视，还有少数中介服务单位违规收费。一些公职人员担当作为不够，敢闯敢试的精神有所弱化，有的为了不出事、宁愿少干事，有的单位“门好进、脸好看、但事还是难办”，等等。我们一定要正视以上问题，始终把人民放在心中最高位置，以功成不必在我的精神境界、以功成必定有我的历史担当，全心全意服务市民，尽心尽力服务企业，努力把我们共同的家园建设得更加美好，使人民政府不负人民的重托！

　　二、2019年主要工作

　　今年是新中国成立70周年，是高质量全面建成小康社会的关键之年，是贯彻习近平总书记对广东重要讲话和对深圳重要批示指示精神、全面推开建设“中国特色社会主义先行示范区、社会主义现代化强国的城市范例”的关键之年，是深圳建市40周年。站在新的发展起点上，我们既面临全面深化改革开放、新一轮科技产业变革和粤港澳大湾区建设等一系列利好叠加新机遇，又面临经济运行稳中有变、变中有忧的新挑战。我们既要树立底线思维、增强风险意识，更要坚定必胜信念、化危为机，抓住并全面用好重要战略机遇期，充分发挥深圳改革先锋、开放先行、创新先发的优势，推动思想再解放、改革再深入、工作再落实，努力实现更高质量、更有效率、更加公平、更可持续的发展。

　　市委六届十一次全会就贯彻落实习近平总书记对广东重要讲话和对深圳重要批示指示精神作了全面部署，发出了当好新时代改革开放尖兵的号召，明确了建设“中国特色社会主义先行示范区、社会主义现代化强国的城市范例”的战略路径，要求做到“十个先行示范”，并决定继续将2019年作为“城市质量提升年”，重点抓好十个方面的工作。我们要按照市委确定的目标任务，全力抓好落实。

　　今年政府工作的总体要求是：高举习近平新时代中国特色社会主义思想伟大旗帜，全面贯彻党的十九大和十九届二中、三中全会以及中央经济工作会议精神，深入学习贯彻习近平总书记对广东重要讲话和对深圳重要批示指示精神，树牢“四个意识”、坚定“四个自信”、坚决做到“两个维护”，围绕实现“四个走在全国前列”、当好“两个重要窗口”，按照省委十二届六次全会和省委“1＋1＋9”工作部署，落实市委“1＋1＋6”系列文件工作安排，始终牢记党中央创办经济特区的战略意图，始终坚持用好改革开放40年成功经验，统筹推进“五位一体”总体布局，协调推进“四个全面”战略布局，坚持稳中求进工作总基调，坚定不移贯彻新发展理念，坚持和加强党的全面领导，坚持全面深化改革，坚持全面扩大开放，坚持以人民为中心，践行高质量发展要求，深入实施创新驱动发展战略，抓住粤港澳大湾区建设重大机遇，增强核心引擎功能，以供给侧结构性改革为主线，深化市场化改革，继续打好三大攻坚战，统筹推进稳增长、促改革、调结构、惠民生、防风险、保稳定各项工作，着力提高发展平衡性和协调性，坚持不懈推进全面从严治党，保持经济持续健康发展和社会大局稳定，朝着建设中国特色社会主义先行示范区的方向前行，努力创建社会主义现代化强国的城市范例，不断推动深圳工作开创新局面、再创新优势、铸就新辉煌，在新时代走在前列、新征程勇当尖兵。

　　今年经济社会发展的主要预期目标是：地区生产总值增长7%左右，地方一般公共预算收入增长6.5%左右，居民收入与经济增长基本同步；居民消费价格涨幅控制在3%以内；新增就业人数8万人，城镇登记失业率控制在3%以内；完成节能减排任务；金融等风险有效防控；新增幼儿园学位2万个、公办中小学学位超过3万个，新增病床3500张、养老床位1000张，建设筹集人才住房和保障性住房8万套、供应3.4万套。

　　为实现上述目标，我们要重点做好以下十个方面的工作：

　　（一）举全市之力推进粤港澳大湾区建设，增强核心引擎功能。牢牢扭住大湾区建设这个“纲”，深度对接港澳所需、深圳所能、湾区所向，携手建设富有活力、国际竞争力的一流湾区和世界级城市群。一是打造前海粤港澳大湾区合作示范区。开展前海总规修编，推动前海合作区扩区，总结拓展“前海模式”。推动出台前海新时代全面深化改革开放方案，探索更多可复制可推广的经验，打造高水平对外开放门户枢纽。争取出台深化深港金融合作创新政策，探索建立本外币一体化的账户体系，设立人民币国际投贷基金，开展金融综合监管试点，扩大粤港澳联营律师事务所业务范围。出台支持港澳青年在前海发展若干措施，建设粤港澳青年创业区。全面推进前海城市新中心建设，启动沿江高速前海段与南坪快速衔接工程、国际交流岛等重大项目规划建设，开工建设前湾会议中心、桂湾公园等公建配套设施，全年完成固定资产投资480亿元以上。二是高水平建设河套深港科技创新合作区。探索建立适应开放创新、融合“一国两制”优势的体制机制，争取在人员物资流动、职业资格准入、标准规则创新、新药品新器械监管等方面先行先试，实现深港科研资金跨境使用，探索建设离岸创新基地。加快合作区空间整备，完善交通配套，推进福田保税区转型升级。三是谋划建设深港商贸旅游消费合作新平台。规划建设口岸经济带，开通莲塘口岸，推进皇岗、沙头角等口岸重建，提升罗湖、深圳湾等口岸环境品质，拓展购物、休闲、文化等功能，建设跨境消费新兴商圈。编制沙头角和罗湖深港国际旅游消费合作区发展规划，推动深港共同开发建设，探索建设特色购物一条街。四是打造大湾区国际综合交通枢纽。强化国际航空枢纽辐射功能，加快第三跑道等规划建设，新增5个以上国际通航城市，推进通用航空发展，加快建设一流的空港经济区。加快盐田港东作业区和西部出海航道二期工程，规划建设深圳港内陆港，推进深港引航互认，推动开设深港海上旅游航线。开通穗莞深城际轨道交通，加快深中通道等对外通道建设，力争深茂铁路深圳至江门段开工，开展深圳至湖南第二高铁、深肇铁路、深圳至南宁铁路等规划研究。高标准规划建设西丽、机场东、光明城、坪山、平湖、深汕等交通枢纽。研究提升深圳站服务功能，引入高铁线路，建设综合交通枢纽，完善地铁接驳，带动片区改造和周边业态提升。五是打造“一带一路”重要支撑区。制定参与“一带一路”建设三年行动方案，加快深越海防合作区、中白物流园建设，探索设立促进境外贸易的服务网点，扩大深圳国际仲裁院海外庭审中心布局。提高工程建设国际化水平，推动更多的工程承包、建筑等企业开拓“一带一路”市场。依托刚启用的“一带一路”环境技术交流与转移中心，规划建设国际化高端环保产业园。出台“走出去”服务体系建设三年行动方案，拓展与国际友好城市、世界先进城市的交流合作，不断提高深圳的国际影响力。六是加快全球海洋中心城市建设。坚持陆海统筹、城海互动，优化城市功能布局，完善海洋功能区划，加强海洋生态保护与整治修复，打造大湾区活力海岸带。推进海洋新城规划建设，启动中欧蓝色产业园和国家南方海洋科学城规划设计，发挥海洋产业发展基金作用，大力发展海洋电子信息、海洋高端装备、海洋生物、海洋金融等产业，规划建设海洋博物馆，办好中国海洋经济博览会。

　　（二）坚持以营商环境改革为重点，狠抓改革攻坚。经济形势越是复杂多变，我们越是要深化市场化改革，以改革的突破来赢得发展的主动，营造更加市场化法治化国际化的营商环境，努力打造全球创新创业和投资发展最佳首选地。一是着力以改革优化营商环境。建设国际一流营商环境改革创新实验区，对标国家营商环境评价指标，对标国际先进城市，动态完善改革举措，持续推进企业开办注销、施工许可、税费缴纳、市场监管、社会信用、跨境贸易等领域改革，继续深化工程建设项目审批“深圳90”改革，推动水、电、气、网等服务便捷化，力争达到相当于世界银行发布的全球经济体营商环境排名前30名的水平。二是着力以改革激发市场主体活力。大力发展民营经济，落实国家、省支持民营经济发展政策措施，继续实施好促进民营经济发展“四个千亿”计划，进一步优化民营经济发展环境，坚决破除各种隐性壁垒，鼓励民营资本进入交通、水利、市政公用事业和战略性新兴产业等领域，鼓励有实力的民间资本参与国有企业改制重组。落实国家更大规模减税降费政策，进一步降低企业社保、融资、用工、用房等成本，大力支持中小微企业发展。开展民营企业梯度培育计划，培育一批民营领军骨干企业和优质上市企业。弘扬企业家精神，构建“亲”“清”新型政商关系，建立健全领导干部联系民营企业制度，满腔热情积极主动做好企业服务。深化科技体制改革，扩大科研人员在技术路线选择、资金使用、团队组建、成果转化等方面的自主权。全面推进区域性国资国企综合改革试验，完善市场化选人用人机制，打造国际一流国有资本投资公司。三是着力以改革解决住有所居问题。坚持房子是用来住的、不是用来炒的定位，以更多的经济手段构建房地产市场健康发展长效机制，加快住房制度改革配套政策落地，推进住宅政策性金融机构改革试点。加大人才住房、安居型商品房、公共租赁住房建设筹集力度，加快光明长圳等一批大型安居社区建设，有序推进老旧小区和城中村的改造。完善住房租赁监管服务平台功能，出台专门政策严控住房和产业用房租金过快上涨。四是着力以改革提高土地节约集约利用水平。深化土地管理制度改革，优化土地资源配置，统筹地上地下空间开发利用，强化供地后续监管。加大成块连片土地清理和整备，加快旧工业区连片升级改造。全年整备土地9平方公里，城市更新供应用地2.5平方公里。坚持“零增量、减存量”，保持查处违法建设高压态势，加快产业类和公共配套类历史违建的分类处理，全年拆除消化各类违法建设2600万平方米以上。五是着力以改革提高政府办事效率。以机构改革为契机，开展政务服务提升专项行动，进一步转变政府职能，深化“放管服”改革，最大限度减少审批事项、办事环节和证明材料，推进网上审批全覆盖，全面优化市、区、街道、社区四级窗口服务。六是着力以改革提升服务企业和人才的水平。完善企业重大危机预警救助和贸易摩擦应对机制，建立企业投诉受理平台和综合服务平台，及时主动向企业推送惠企政策信息，让企业找得到、看得懂、用得上。规范涉企执法检查事项，推行执法事前干预、主动指导、及时提醒制度。创新人才服务，完善人才综合服务平台，促进粤港澳大湾区人才更加便捷流动，推动更多科研机构和企业开展人才自主评价。全年新引进全职院士10名，新认定高层次人才1000名以上。

　　（三）坚持把创新作为城市发展主导战略，加快建设国际科技产业创新中心。着力强化协同创新、集成创新、应用创新优势，深入实施高新技术产业高质量发展“七大工程”，积极参与广深港澳科技创新走廊建设，争创综合性国家科学中心，大力发展壮大新动能，努力在全球新一轮科技产业变革中发挥主力军作用。一是建设世界一流的光明科学城。对标最高标准、最高水平，加快推进材料基因组等大科学装置建设，前瞻布局重大创新平台和创新孵化器。加大土地整备力度，加快重点项目和配套设施建设。二是加快补齐基础研究短板。加大基础研究投入力度，确保基础研究投入比例不低于财政科技专项资金的30%。设立深圳市自然科学基金，实施一批重大科技专项。高起点规划建设西丽湖国际科教城。推进未来网络试验设施等重大科技基础设施建设，争取国家第三代半导体技术创新中心等落户深圳，做实诺贝尔奖科学家实验室。全年新增国家、省重点实验室等各类创新载体100家以上。三是打造新兴产业发展高地。大力发展新一代信息技术、高端装备制造、绿色低碳、生物医药、数字经济、新材料、海洋经济等战略性新兴产业。开展高新技术企业增量提质行动，新增国家级高新技术企业2000家以上，培育更多拥有颠覆性技术的科技企业。推动高新区创新发展，加快高新北区升级改造，高水平规划建设坪山高新区。推动国家生物产业基地、大梧桐新兴产业带等建设。创建国家军民融合创新示范区。四是完善全过程创新生态链。促进科技与金融深度融合，用好天使投资引导基金，探索设立科技小额贷款、科技金融租赁和科技保险公司。建设国家知识产权强市，落实知识产权保护条例，推进知识产权质押融资和证券化，构建快速授权、确权、维权的保护机制。举办“全球青年创新集训营”活动，继续办好高交会、电子信息博览会和IT、BT领袖峰会。

　　（四）做强做优实体经济，打造经济高质量发展的示范区。落实“六个稳”部署，贯彻“巩固、增强、提升、畅通”方针，坚持以供给侧结构性改革为主线不动摇，坚持质量第一，实施质量强市战略，持续加强深圳质量、深圳标准、深圳品牌、深圳设计、深圳信誉建设，构筑更具竞争力的现代产业体系，率先推动质量变革、效率变革、动力变革，进一步强化深圳全国经济中心城市的集聚辐射功能。一是推动制造业高质量发展。推进华星光电T7、亚太卫星、大族激光智能制造、全普微激光等重大项目建设，打造新能源汽车等先进制造业集群，创建医疗器械等国家制造业创新中心，着力引进一批投资10亿元以上的先进制造项目。实施新一轮重大技术改造升级工程，推进一批工业强基补短板项目，进一步扩大工业投资。二是持续打造深圳标准、深圳品牌。把标准和品牌建设作为制造业提质增效的重要抓手，建设标准国际化创新型城市，推出一批更高水平的团体标准、行业标准、企业联盟标准，参与或主导制定国际国内标准400项以上，新建5个技术标准创新基地。大力开展品牌推广计划，创建一批品牌示范区，推动黄金珠宝、眼镜、钟表、服装、家具等行业品牌走向世界。三是提升金融中心集聚辐射功能。支持深交所创新发展，加快建设创新资本中心、国际风投创投中心和财富管理中心，新引进持牌金融机构10家以上，新培育一批上市公司。大力发展科技金融、绿色金融、黄金金融。加大对中小微企业的信贷支持力度，试点政策性小额贷款保证保险。深化互联网金融风险专项整治，严厉打击非法集资、金融诈骗等违法行为。落实支持金融人才发展政策措施，发挥好资本市场学院等平台作用。四是打造生产性服务业高地。强化现代物流枢纽功能，出台促进供应链金融发展的意见，大力发展供应链管理、电商物流、智能仓储等新业态。壮大会展经济，制定促进会展业发展政策措施，建成并投入使用国际会展中心，完善交通等周边配套服务。提升会计审计、法律服务、商务咨询等专业服务业发展水平。新增一批检验检测、技术转移等公共服务平台，加快深圳医疗器械审评认证中心等建设，筹建中国计量院技术创新研究院。五是着力促进内外需协调发展。扩大高质量有效投资，用好政府专项债，加大基础设施、重点区域、公建配套等投资力度，全年固定资产投资约7000亿元、增长13%左右。着力释放消费潜力，大力发展新零售等新业态，开展商业名街建设行动，建设国际消费中心城市。实施外贸稳增长计划，推动出口市场多元化，扩大关键设备、核心零部件、平行汽车等进口。大力发展跨境电商等新业态，优化保税区管理体制。落实省“外资10条”，提高利用外资的质量和水平。

　　（五）打造国际一流智慧城市，抢抓智能时代发展先机。充分发挥深圳信息产业优势，加快布局人工智能技术和产业，率先壮大智能经济、建成数字政府、迈入智能社会。推动实现“一图全面感知”“一号走遍深圳”“一键可知全局”“一体运行联动”“一站创新创业”“一屏智享生活”，培育新一轮高质量发展的新动力。一是构建全球领先的智能化信息基础设施。加快高速宽带网络工程建设，率先开展5G商用试点，超前布局物联网、智能网联汽车等新型基础设施，加快超算中心二期建设。完善大数据基础设施，建成政府管理服务指挥中心和城市大数据中心。推进全面感知体系工程建设，加快城市基础设施智能化改造。二是建设智能经济发展先导区。率先打造数据驱动、人机协同、跨界融合、共创分享的智能经济形态，出台新一代人工智能发展行动计划和芯片产业发展政策，建设人工智能制造业创新中心。加快突破芯片、算法、感知等关键技术，大规模拓展人工智能在先进制造业、公共服务、社会治理等领域的应用场景。建设一批工业互联网平台和智能工厂，推进制造业数字化、网络化、智能化升级改造。三是打造一流数字政府。组建市政务服务数据管理局，强化电子政务服务、大数据管理的有机融合和高效协同。全面推广“i深圳”APP，努力实现95%以上个人事项和60%以上法人事项移动办理。新推出“秒批”服务和“不见面审批”事项各200项。整合全市政务信息系统，实现数据标准化采集、动态化更新、规范化使用，推动数据开放共享，强化信息安全和隐私保护。四是大力推广智慧民生服务。突出用户体验、强化政企联动，拓展智慧交通、智慧医疗、智慧教育等应用。推行智能交通信号控制系统、智慧停车和多功能智能杆。全面实现医院和社康中心检查结果互认互通。完善教育云平台，促进优质教育资源开放共享。推进生态环保、公共安全、市场监管等智慧监测预警，实现实时监测、动态监管、联动执法。

　　（六）坚决打好污染防治攻坚战，全面提升生态文明建设水平。深入践行绿色发展理念，继续抓好中央、省环保督察反馈意见整改，以更大力度推进水体、大气、固废、土壤、噪声等污染防治，努力让城市水更清、天更蓝、环境更优美，建设美丽深圳。一是全力开展“水污染治理决战年”工作。压实河长、湖长、湾长责任，统筹执法措施、管理措施、工程措施、技术措施，确保全面消除全市黑臭水体，实现深圳河、观澜河、龙岗河、坪山河考核断面水质稳定达到地表水Ⅴ类水质，推动茅洲河水质明显改善。坚持“厂、网、河”一体推进，新建扩建和提标改造23座水质净化厂，新增污水处理能力40万吨/日。开展污水管网查漏补缺，全面完成剩余4000多个小区雨污分流改造，实施化粪池、隔油池、管道沉积物清理专项行动。加强交叉执法、联合执法，完成“散乱污危”企业综合整治。深入开展汽修、农贸市场等涉水污染源专项治理。推进电镀等工业企业入园，加快江碧环境生态产业园建设。完善全流域一体化生态补水系统，推进防洪、防潮和排涝项目建设，推动笔架山河等暗渠复明，启动“万里碧道”深圳行动计划，坚持源头治理、全面截污、集中处理、达标排放、清淤补水、岸绿景美、便民配套的工作思路，努力将全市河湖打造成为鱼翔浅底、水清岸绿的美好生态空间。二是深入实施“深圳蓝”可持续计划。全面推广国Ⅵ标准车用燃油，大力推广应用新能源汽车，加快工程车电动化更新应用。加强机动车排放监管，淘汰老旧车5万辆，加快充电桩建设，建立动力电池回收利用体系。加强挥发性有机物、工地扬尘、餐饮油烟、露天烧烤等污染治理。推进船港污染防治，实现靠港船舶全部使用低硫燃油。加强排污企业的监管和治理，努力提高空气质量。三是着力破解垃圾处理难题。新增垃圾焚烧处理能力1.1万吨/日、污泥处置能力800吨/日，加快坪山、龙华等生活垃圾处理设施规划建设，完成400座垃圾中转站升级改造，加强垃圾填埋场整治。加快环保产业园区建设，提高建筑、医疗等废弃物处置能力。实施全面推进生活垃圾强制分类行动计划，落实“八个一”的工作措施，实现厨余垃圾分类全覆盖，生活垃圾回收利用率达30%以上，努力让垃圾分类成为新风尚。四是持续推进城市净化绿化美化。创新道路保洁和绿化维护管养体制机制，创新城中村管理服务方式，推进物业管理全覆盖，完成600个以上城中村综合治理。继续提升森林生态质量，建成100个以上花景项目，新建改造提升40个公园，举办粤港澳大湾区深圳花展。优化城市公共空间，完善运动、直饮水、母婴室、公共厕所、无障碍通道等便民设施，让市民更好亲近自然、健康生活。

　　（七）深入实施“东进、西协、南联、北拓、中优”发展战略，推动城市高水平发展。我们要下绣花功夫建设管理好城市，系统优化城市功能，推动全市域均衡发展和各区错位发展，全面提升城市发展能级。一是推动城市一体化发展。主动融入全省“一核一带一区”区域发展新格局，加大特区一体化攻坚力度，优先将教育、医疗、文化、科研等重大项目布局原特区外。打造坂雪岗科技城、北站商务中心区、笋岗－清水河片区、西丽高铁新城、坪山中心区、坝光国际生物谷、盐田中心区等一批城市新空间，加快华富村、湖贝、新桥东等旧城区更新改造，带动周边基础设施完善。高起点规划建设深汕特别合作区，推动深汕高铁、深汕第二高速等项目尽快开工，加快小漠国际物流港、深东大道、深汕高级中学、深汕医院、深汕环境科技产业园等项目建设，打造现代化滨海新城、产业新城。二是高标准建设“城市新客厅”。坚持用最先进的理念、最好的技术、最优的方案、最精细的管理，加快前海城市新中心、深圳湾超级总部基地、香蜜湖新金融中心、光明科学城、大运新城、宝安中心区等片区规划建设，强化重点片区开发建设指引，制定城市设计指南和工程建设导则，打造国际一流的时代精品、城市杰作。三是全面加快基础设施建设。推动轨道交通三期四期14条线路建设提速，推进轨道交通五期规划，启动东部地区至机场轨道快线前期工作。建成南坪三期、龙澜大道等重要通道，加快外环高速、东部过境高速、坪盐通道、坂银通道、春风隧道、沙河西路快速化改造等项目建设，新开工黄木岗交通枢纽、梅观高速清湖南段市政化改造等工程，启动机荷－惠盐高速改扩建、龙大高速市政化改造、皇岗路快速化改造等前期工作，加快完善“十横十三纵”高快速路网体系。优化新增公交线路85条以上，打通断头路10条，新改扩建自行车道约300公里，推进重点拥堵路段和热点片区交通综合整治。新增优质饮用水入户小区200个。加强各类市政工程建设的统筹，切实减少道路反复开挖，强化文明施工管理，尽量减少施工扰民，努力做到工完场清。四是创建安全发展示范城市。制定城市安全发展实施意见，强化科技兴安、制度强安，深入落实安全生产责任制，提升全民安全文明素质。完善风险分级分类管控机制，持续加大消防、建设工程、危化品、特种设备等专项整治。制定规范电动单车、泥头车管理规定，严厉打击逆向行驶、冲闯红灯、违章变道等违法行为。建立高效科学的灾害防范体系，加强专业应急队伍建设，开展应急实战盲演，提升应急救援和处置能力。持续推进平安深圳建设，深入开展扫黑除恶专项斗争，提升市民安全感。

　　（八）建设全球区域文化中心城市，增强城市文化影响力。深入实施“文化创新发展2020”，培育和践行社会主义核心价值观，大力发展文化事业和文化产业，开展文化旅游品质提升行动，打造国际文化创新创意先锋城市，让文化软实力成为特区新的竞争力。一是高水平建设文体设施。推进深圳歌剧院、科技馆、国家博物馆深圳馆等新十大文化设施规划建设，开工建设美术馆新馆、中国红树林博物馆、深圳湾文化广场和宝安公共文化艺术中心，建成坪山文化中心等设施，完善提升南头古城等十大特色文化街区，筹建深圳工业博览馆新馆。加快嶂背郊野公园、海滨生态体育公园、宝安滨海文化公园等主题公园建设。新建80个以上便民利民健身场所。二是大力发展文体事业。围绕新中国成立70周年、建市40周年等主题举办系列文化活动，继续开展市民文明素养提升行动。实施文艺精品创作和文艺名家推广计划，打造特色“城市文化菜单”。继续办好文博会、读书月、“一带一路”国际音乐季、来深青工文体节、公园文化季等文化活动。进一步加强文物保护，挖掘、活化并利用好历史建筑和文化街区。办好WTA年终总决赛、男篮世界杯深圳赛区比赛、海峡两岸学生棒球联赛总决赛等赛事，鼓励社会力量举办高水平赛事。推进青少年足球试验区建设，积极创建全民运动健身模范市。三是推动文化产业创新发展。出台文化产业创新发展和创意设计高质量发展的政策措施，做大时尚设计、工业设计、建筑设计等产业，培育发展影视、动漫、数字出版等产业，擦亮华侨城创意文化园、大浪时尚创意小镇等品牌。筹办创意设计馆，开业运营前海深港设计创意产业园，提升深圳设计周、深圳时装周、深港设计双城展等国际知名度，打造更具魅力的设计之都。四是建设世界级滨海旅游度假区。推进国家全域旅游示范区建设，高标准打造大小梅沙、西涌等滨海旅游带，创建大鹏半岛国家级海洋公园，建设一批精品旅游设施。加快中国邮轮旅游发展实验区建设，争取实施更加便捷的通关、退税、过境免签等政策，持续提升旅游服务品质。

　　（九）落实精准脱贫攻坚战部署，坚决完成对口支援和扶贫协作任务。贯彻乡村振兴战略，坚持产业扶贫、造血式扶贫，全力推动先富帮后富、携手奔小康，全年安排帮扶资金28亿元。继续做好援疆援藏工作，建成喀什深圳产业园三期工程，推进富民安居工程和人工草场、牦牛养殖等项目。扎实推进对口广西百色、河池扶贫协作，加强劳务输出、农产品、产业园区、旅游等方面合作，继续推进教育扶贫、医疗扶贫。认真做好全面对口帮扶河源、汕尾工作，抓好深河、深汕产业共建园区建设，推动建设好深河人民医院、深汕中心医院等民生项目，精准推进323个相对贫困村稳步脱贫。扎实推进四川甘孜、重庆巫山、贵州毕节、江西寻乌等帮扶工作。此外，积极推动与哈尔滨对口合作，共同加快哈深产业合作园规划建设。

　　（十）着力建设幸福深圳，营造共建共治共享的社会治理新格局。全年计划完成十个方面40项民生实事和一批民生微实事，安排九大类民生支出2709亿元，其中教育、医疗支出分别增长11%和13%。一是促进基础教育优质均衡发展。优先发展教育事业，出台高质量推进教育现代化的实施意见，扩大和优化基础教育学位供给。坚持立德树人，提升学生身心健康水平和艺术素养，培养创新思维、劳动意识和服务精神。打造高素质专业化创新型教师队伍，积极推进中小学教师职称制度等改革，实施中小幼教师学历提升计划。持续深化幼儿园托底改薄专项行动，提高学前教育普惠优质发展水平。加强婴幼儿早期教育服务监管，实现新型公办园和普惠性民办园在园儿童占比80%左右。新改扩建中小学校18所，推动校内午餐午休率达到90%左右。规范校外培训机构发展，切实减轻中小学生课外负担。鼓励社会力量举办高水平国际学校，进一步提升民办教育质量。发展特殊教育。二是推进高等教育高水平发展。支持深圳大学、南方科技大学、香港中文大学（深圳）、哈尔滨工业大学（深圳）等建设高水平大学。建成深圳北理莫斯科大学校区，推进清华大学深圳国际研究生院、中山大学深圳校区、深圳技术大学等建设，加快筹建中科院深圳理工大学。加强与国际一流大学交流合作，研究推进音乐学院、师范学院和创新创意设计学院建设。支持深圳职业技术学院、深圳信息职业技术学院建设国际一流职业院校。三是深入推进“健康深圳”建设。加快高水平医院建设，继续实施“三名工程”，打造国际化医疗高地。新增三甲医院2家、三级医院4家，积极发展国际化的医疗健康管理机构。建成南方医科大学深圳口腔医院和市人民医院内科大楼改扩建等项目，加快新华医院、第二儿童医院、中山大学附属七院二期、平湖医院、吉华医院、大鹏医院和宝安人民医院新楼等项目建设，推动建设急救血液信息三中心、国家心血管疾病临床医学深圳中心。加大先进医疗设施等布局，加快质子肿瘤治疗中心建设。积极筹建南科大医学院、港中大（深圳）医学院。全面加强社康中心标准化建设，新增社康中心30家以上。完善全科医生激励机制，开通家庭医生服务热线，实现基层诊疗量占比提高至45%以上。深化公立医院人事薪酬制度、医保支付方式等改革，全面实施药品、医用耗材和设备集团化采购。实施公共卫生强化行动，新增少儿近视防治、脊柱侧弯筛查、疫苗智能冷链建设服务，完善心理健康服务体系。四是打造市民满意的食品安全城市。深入实施食品安全战略，加强供深食品标准体系建设和认证工作，推进供深食品基地、“熟食中心”、长者饭堂等建设。推动农产品市场升级改造，完善生产、流通、消费等全链条溯源监管。实施阳光智慧餐饮工程三年计划，全面推行“互联网＋明厨亮灶”，狠抓工厂、学校食堂和网络送餐等质量安全监管，制定学生营养餐深圳标准。出台进一步加强药品安全监管措施，用最严格的标准强化疫苗、血液制品等安全监管。五是强化基本民生保障。把稳就业摆在更加突出位置，加强高校应届毕业生、户籍困难人员等重点群体就业服务。做好困难群众和弱势群体的兜底保障工作。推动市社会福利救助综合服务中心等项目建设，新增一批老年人日间照料中心，探索建立老年人长期护理保险制度，完善综合养老服务体系。营造儿童健康成长的良好环境，建设儿童友好型城市。积极创建无障碍城市，推进残疾人服务“四个一”工程建设。六是加强社会治理。出台完善社区治理实施方案，整合基层执法、管理、服务等人员力量，推进专业社工管理体制改革。加强劳资纠纷排查治理，积极构建和谐劳动关系。扎实做好双拥、民防、国防动员等工作。积极发展公益慈善事业，继续办好中国慈展会。推广北站社区、同胜、水围、罗湖物业管理等基层治理新模式，拓展来深建设者参与社会治理的途径和方式，努力把深圳建设成为全国最安全稳定、最公平公正、法治环境最好的城市之一。

　　新的一年，面对新机遇、新挑战，我们要出色地完成好全年工作任务，必须全面加强政府系统党的建设，坚持用习近平新时代中国特色社会主义思想武装头脑、指导实践、推动工作，自觉在思想上政治上行动上同以习近平同志为核心的党中央保持高度一致，全面提升履职能力和水平，不折不扣贯彻落实好党中央国务院、省委省政府和市委的工作部署。必须坚持依法行政，自觉接受市人大及其常委会的法律监督、工作监督和市政协的民主监督，坚决执行市人大及其常委会各项决议决定，坚持重大事项向市人大报告、向市政协通报。进一步提高人大代表建议和政协提案的办理水平，认真听取各民主党派、工商联、无党派人士和各人民团体的意见，高效回应社会关切。必须厉行勤俭节约，牢固树立过紧日子的思想，进一步优化财政支出结构，严控一般性支出，确保今年一般性新增项目支出预算经费压缩5%，“三公”经费压缩3%，会议费压缩20%，切实提高财政资金使用效益。必须坚持不懈抓落实，坚定不移纠“四风”、树新风，力戒形式主义、官僚主义。加强调查研究，严格规范督查、检查、考核等事项，整治过度“痕迹管理”等问题，持续提升政府执行力，始终保持苦干实干、能干善干的实践品格。必须加强勤政廉政建设，严格落实中央八项规定及实施细则精神，坚决惩治各类腐败行为。加大审计监督、政务公开力度，加强廉政风险防控。优化绩效考核，激励公职人员担当作为，努力建设人民满意的廉洁政府、阳光政府、法治政府。

　　各位代表，深圳经济特区发展已走过千山万水，但仍需跋山涉水。让我们更加紧密地团结在以习近平同志为核心的党中央周围，在省委省政府和市委的坚强领导下，解放思想、真抓实干，闻鸡起舞、日夜兼程、风雨无阻，奋力朝着建设中国特色社会主义先行示范区的方向前行，努力创建社会主义现代化强国的城市范例，在新时代走在前列、新征程勇当尖兵，为实现“两个一百年”奋斗目标和中华民族伟大复兴的中国梦作出新的更大贡献，以优异成绩庆祝中华人民共和国成立70周年和深圳建市40周年！
